# Highly activated p53 contributes to selectively increased apoptosis of latently HIV-1 infected cells upon treatment of anticancer drugs

**DOI:** 10.1186/s12985-016-0595-2

**Published:** 2016-08-16

**Authors:** YoungHyun Shin, Hoyong Lim, Byeong-Sun Choi, Kyung-Chang Kim, Chun Kang, Yong-Soo Bae, Cheol-Hee Yoon

**Affiliations:** 1 Division of AIDS, Center for Immunology and Pathology, Korea National Institute of Health, 187 Osongsaengmyeong 2-ro, Osong-yeup, Cheongju, Chungbuk 363-951 South Korea; 2 Department of Biological Sciences, Sungkyunkwan University, Suwon, Republic of Korea

**Keywords:** Latently HIV-1 infected cells, Tumor suppressor p53, Anticancer drugs, Apoptosis

## Abstract

**Background:**

Despite the successful inhibition of human immunodeficiency virus type 1 (HIV-1) replication by combination antiretroviral therapy, cells latently infected with HIV-1 remaining in patients are a major obstacle for eradication of HIV-1 infection. The tumor suppressor factor p53 is activated by HIV-1 infection, and restricts HIV-1 replication. However, a therapeutic strategy based on p53 activity has not been considered for elimination of latently infected cells.

**Methods:**

Apoptotic cells were analyzed using flow cytometry with anti-annexin A5-FITC Ab and PI staining upon treatment of anticancer drugs. The expression and activation of p53 and apoptotic molecules in latently HIV-1-infected T cells were compared using Western blot analysis. The role of p53 in the anticancer drug treatment-induced apoptosis of cells latently infected with HIV-1 was determined by knock-down experiment using siRNA against p53.

**Results:**

Upon treatment with 5-fluorouracil (5-FU), apoptosis was increased in latently infected ACH2 cells encoding competent p53 compared with uninfected parent A3.01 cells, while the apoptosis of latently infected p53 null J1.1 cells was less than that of uninfected cells. Treatment with 5-FU increased the levels of cleaved caspase-3 and PARP in ACH2 cells compared with uninfected and latently infected p53 null J1.1 cells. The levels of expression and activation of p53 were higher in both latently infected ACH2 and NCHA2 cells than in uninfected cells. Furthermore, the activation levels of p53 in both cells were further increased upon 5-FU treatment. Consistent with p53 status, apoptosis was markedly increased in ACH2 and NCHA2 cells compared with uninfected and latently infected J1.1 cells upon treatment with other anticancer drugs such as doxorubicin and etoposide. Inhibition of p53 in cells with latent HIV-1 infection diminished apoptosis upon 5-FU treatment.

**Conclusion:**

Evidence described here indicate that when treated with anticancer drugs, apoptosis of cells with latent HIV-1 infection was increased via the p53 activation pathway and may provide information for application of anticancer drugs to selectively eliminate HIV-1 reservoirs.

## Background

Persistent human immunodeficiency virus type 1 (HIV-1) infection causes a progressive loss of CD4+ T cells, followed by acquired immunodeficiency syndrome (AIDS) [1, 2]. Although highly active antiretroviral therapy (HAART) can improve the life-span of HIV-1 infected patients by successful suppression of the HIV-1 replication, HAART is not able to eliminate the latent reservoir established early during infection [3]. Accordingly, latent reservoirs of HIV-1 infection have importantly been recognized as a major barrier to HIV-1 eradication [4, 5].

Numerous strategies for the elimination of latent HIV-1 infection have been investigated. These strategies include intensive ART regimens, transplantation of CCR5-deleted bone marrow, and reactivation of HIV-1 expression. One of the most innovative strategies aims to awaken the latent virus and thereby increase the killing of the latent infection reservoir by the immune system, known as “shock and kill”, and uses T-cell activating cytokines (IL-7), T-cell receptor signaling pathway activators (bryostatin and prostratin as a protein kinase C activator), histone deacetylase inhibitors (valproic acid, vorinostat (suberoylanilide hydroxamic acid)), DNA methylase inhibitors, and disulfiram [6]. Although these reactivating strategies have recently been shown to increase viral RNA in patients, reduction of reservoir size has not been consistently successful.

Tumor suppressor p53 plays a key role in the stimulation of cell apoptosis and growth arrest through a cooperative signaling network of genotoxic stress caused by treatment with anticancer drugs, irradiation, ultraviolet light, and glucose starvation [7]. HIV-1 infection also induces genotoxic stresses linked to p53 activation in CD4+ T cells by integration mediated- dsDNA strands break, secretion of type I interferons and expression of HIV-1 accessory proteins Vpr/Vpu, which may be considered as intracellular markers of HIV-1 infection [8–11]. The p53 activation by HIV-1 infection also plays an important role in host-restriction mechanisms against HIV-1 replication, which suppress long terminal repeat (LTR)-mediated viral transcription through Tat modulation [12].

With regard to apoptosis of HIV-l infected cells, an earlier report showed that latently HIV-l infected H9, J1.1 and U1 cells that are p53 null were more resistant to apoptosis than their uninfected parent cells under treatment with H_2_O_2_ and staurosporine (STS) which leads to an increase in oxidative stress rather than genotoxic stress [13]. In a recent study, ACH2 and U1 cells showed induction of HIV-1 reactivation by an alternative replication program (ARP) during cell apoptosis after cytotoxic drug treatment [14]. Although these studies suggested that apoptosis might be an important characteristic of latent HIV-1 infection, these data might be missed by an examination of p53 status and comparative analysis of the apoptotic ratio between the latently infected and uninfected cells. Currently, the important roles of p53 have been discussed in HIV-1 infection. However, the p53-mediated apoptosis of latently infected cells by treatment of anticancer drugs has not been considered in developing strategies for eliminating cells latently infected with HIV-1. Herein, we hypothesize that apoptosis may be more sensitively induced in latently infected cells encoding competent p53 than uninfected cells upon treatment with anticancer drugs. We found that anticancer drugs greatly enhanced apoptosis of latently infected cells compared with uninfected cells, and knockdown of p53 significantly diminished the apoptosis. Our data suggest that anticancer drugs may be useful agents for selectively purging HIV-1 reservoirs.

## Methods

### Cells, anticancer drugs and antibodies

A3.01 and cell lines latently infected with HIV-1 (A3.01-derived ACH2 and Jurkat-derived J1.1) were obtained from the NIH AIDS Research and Reference Reagent Program. Latently infected A3.01-derived NCHA2 cells were established in our laboratory [15]. Cells were cultured as previously described [7]. Briefly, cells were maintained in RPMI medium (Gibco-BRL) supplemented with 1 % L-glutamine, 1 % penicillin–streptomycin, and 10 % (v/v) heat-inactivated fetal bovine serum (FBS; Gibco-BRL) in an incubator at 37 °C under an atmosphere of 5 % CO_2_. The anticancer drugs, 5-fluorouracil (5-FU), doxorubicin, and etoposide were purchased from Sigma-Aldrich (USA) and treated with the concentrations indicated in previous reports [16–19]. Antibodies (Ab) against caspase-3, cleaved caspase-3, PARP, Bax, puma, phosphor-S^15^-p53, and β-actin were obtained from Cell Signaling Technology (USA). Anti-p53 antibodies (DO-1) were obtained from Santa Cruz Biotechnology (USA).

### Flow cytometric analysis of apoptosis

Early apoptotic (annexin A5-FITC-positive, propidium (PI)-negative) and late apoptotic (annexin A5-FITC-positive, PI-positive) cells were analyzed using flow cytometry with anti-annexin A5-FITC Ab and PI staining [20]. Briefly, cells treated with 5-FU for 24 h were then incubated with anti-annexin A5-FITC Ab and PI (Calbiochem) for 10 min at room temperature. The stained cells were detected using a FC500 flow cytometer and analyzed with RXP1.0 software (Beckman Coulter). The analysis of sub-G1 cell populations has been described previously [19, 21]. Briefly, cells were incubated with drugs for 36 h. Cells were then fixed with 70 % ice-cold ethanol and incubated overnight at 4 °C. Fixed cells were incubated with 100 μg RNase A at 37 °C for 1 h and stained with 50 μg/ml of PI. For measurement of intracellular DNA content, at least 10,000 events were analyzed. All experiments were performed independently at least three times.

### Western blot analysis

Western blot analysis was performed as previously described [22]. Briefly, cells were treated with an anticancer drug, 5-FU, doxorubicin, or etoposide for 12 h and then harvested in RIPA buffer (Cell Signaling Technology) containing a complete EDTA-free protease and phosphatase inhibitor cocktail (Roche). Forty μg of protein extract was subjected to 10 % sodium dodecyl sulfate polyacrylamide gel electrophoresis (SDS–PAGE) and transferred onto polyvinylidene difluoride membranes (Millipore). The membranes were incubated with appropriate antibodies and then visualized by chemiluminescence using Image Lab software (Bio-Rad) according to the manufacturer’s protocols.

### Knockdown and ectopic expression of p53

Five synthesized p53 siRNAs were purchased from Invitrogen. Their sequences were as follows: No. 1 (VHS40366): CCA UCC ACU ACA ACU ACA UGU GUA A, No. 2 (VHS40367): CCA GUG GUA AUC UAC UGG GAC GGA A, No. 3 (HSS110905): GCU UCG AGA UGU UCC GAG AGC UGA A, No. 4 (HSS186390): GAG UGG AAG GAA AUU UGC GUG UGG A, and No. 5 (HSS186391): CCG CCU GAG GUU GGC UCU GAC UGU A. Knockdown of p53 was performed by electroporation as described previously [12]. Briefly, 1 × 10^7^ cells were electroporated with 500 pmole of control siRNA (si-con; Invitrogen) or p53 siRNAs in 0.4 cm cuvettes at 960 μF and 250 V. The ectopic expression of p53 in J1.1 cells was performed by electroporation using a Nucleofector™ kit X (Lonza) with 5 μg of pcDNA3- Flag-tagged p53 according to the manufacturer’s protocol [22].

### Statistical analysis

The data are expressed as their mean value ± standard deviation (SD). The data were compared using Student *t* tests and *p* <0.05 was considered a significant difference.

## Results

### Distinct sensitivity of cells latently infected with HIV-1 to apoptosis upon 5-FU treatment

**Fig. 1:**
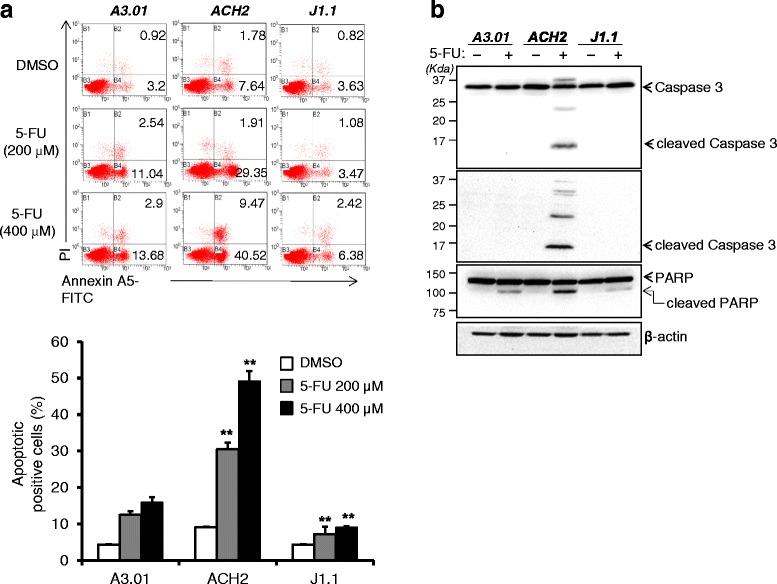
5-FU treatment-induced apoptosis of cells latently infected with HIV-1. **a** The cells were treated with 5-FU at the indicated concentration for 24 h. After treatment, the cells were measured using flow cytometry. The number of apoptotic cells is shown as a percentage in each plot (*upper* panel). Total apoptotic cells are shown graphically with statistical analysis. The data are shown as mean ± SD (*n* = 3). **p <0.01 compared with uninfected A3.01 cells (*lower* panel). **b** The cells were treated with 400 μM of 5-FU for 48 h, and then lysed with RIPA buffer. Western blotting was used to analyze the protein levels from the cell lysates using antibodies against caspase 3, cleaved caspase 3, PARP and β-actin as a loading control

Although numerous previous investigations have shown apoptosis of cells infected with HIV-1, apoptosis of latently infected cells is as yet little known. To address this issue, we compared the apoptotic ratio between latently infected cells and uninfected cells in the presence of anticancer drug-induced genotoxic stress. As shown in Fig. 1a, using flow cytometry analysis, two cell lines latently infected with HIV-1 (ACH2 and J1.1) and an uninfected cell line (A3.01) all showed increased apoptosis after treatment with 5-FU. Notably, we found greatly increased apoptosis in latently infected ACH2 cells compared with other cells examined. However, the other latently infected cells, J1.1, showed slightly decreased level of apoptosis in comparison with uninfected A3.01 cells upon 5-FU treatment. These phenomena were observed in both early and late apoptosis (Fig. 1a). The proteolysis of caspase-3 and its substrate PARP occurring in cells undergoing apoptosis was dramatically increased in latently infected ACH2 cells by 5-FU treatment, whereas proteolysis was barely detected in uninfected A3.01 and latently infected J1.1 cells (Fig. 1b). These data indicate that after 5-FU treatment, cells latently infected with HIV-1 have a distinctive cell fate based on their distinct cellular machinery.

### Tumor suppressor p53 is linked to the 5-FU treatment-induced apoptosis of cells latently infected with HIV-1

**Fig. 2:**
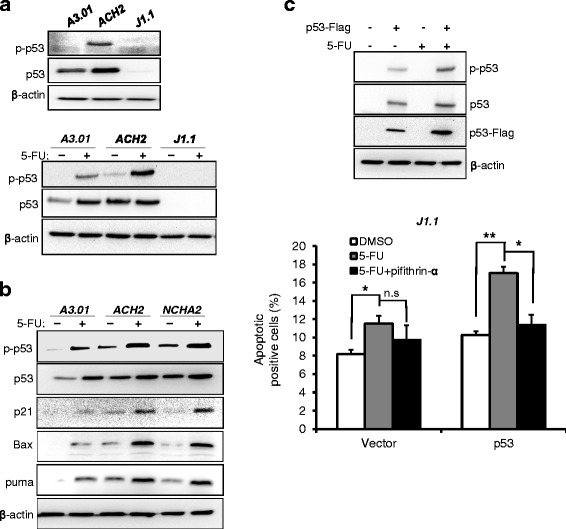
Expression of p53 and its downstream proteins in cells latently infected with HIV-1. **a** The expression levels of p53 and phosphorylated p53 at Ser 15 (p-p53) in latently infected and uninfected cells were measured in the absence (*upper* panel) or presence of 200 μM of 5-FU for 12 h (*lower* panel). **b** The levels of expression of p53 and its downstream proteins were measured in latently infected ACH2 and NCHA2 upon treatment of 200 μM of 5-FU for 24 h. **c** Western blotting was used to analyze the p53 and p-p53 levels in J1.1 cells electroporated with p53 expression plasmid upon 5-FU treatment (*upper* panel). Apoptosis of the cells was measured as Fig. 1a. Total apoptotic cells are shown graphically with statistical analysis. The data are shown as mean ± SD (*n* = 3). *p <0.05, **p <0.01 compared with cells untreated with the drug

Next, we sought to determine which cellular modulator is associated with the distinctive fate of latently infected cells during 5-FU-induced apoptosis. Previous studies showed that p53 was activated in cells acutely infected with HIV-1. Consistent with acute infection, the expression of p53 was increased in latently infected ACH2 cells compared with their parent A3.01 cells. Notably, the phosphorylation level of p53 was significantly enhanced in ACH2 cells despite the lack of a specific stimulus, which might be caused by latent infection-induced stresses (Fig. 2a, upper panel). However, the expression of p53 was not detected in latently infected J1.1 cells originating from the Jurkat cell line that is known as p53 defective. Moreover, the increased levels of total and phosphorylated p53 in ACH2 cells were further enhanced by 5-FU treatment compared with those in A3.01 cells, while J1.1 cells showed no expression of p53 despite this stimulus (Fig. 2a, lower panel). To evaluate p53-mediated cell apoptosis in latently infected cells upon genotoxic stress, the molecules downstream of p53 in latently infected ACH2 and NCHA2 cells that express the wild-type p53 were examined in the presence and absence of 5-FU. The levels of expression of Bax, puma, and p21 which are well known as p53 target genes, were highly elevated together with the levels of p53 activation in ACH2 and NCHA2 cells when cells were treated with 5-FU, while the increased levels were modest in uninfected cells (Fig. 2b). To confirm the p53-linked cell apoptosis in latently infected cells, p53 null J1.1 cells were ectopically expressed with p53 upon 5-FU treatment. The cells ectopically expressed with p53 showed increased apoptosis upon 5-FU treatment compared with mock expression. Furthermore, the treatment of the p53 inhibitor, pifithrin-α, in the cells showed diminished apoptosis compared with vehicle-treated J1.1 cells expressed with p53 (Fig. 2c). Unpredictably, the levels of apoptosis induced by ectopic p53 expression were lower than those of the p53 competent cells latently infected with HIV-1. These data indicate that the cell fate linked to apoptosis in cells latently infected with HIV-1 was likely to be dependent on the status of p53 upon treatment with anticancer drugs such as 5-FU.

### Induced genotoxic stress and increased apoptosis of cells latently infected with HIV-1 upon anticancer drug treatment

**Fig. 3:**
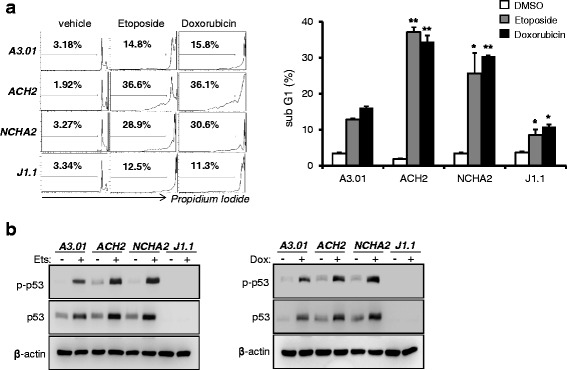
Anticancer drugs increased the death of cells latently infected with HIV-1. **a** Cells were treated with etoposide (5 μM) and doxycycline (0.5 μM) for 36 h. The sub-G1 population (dead cells) of cells was determined by flow cytometry after PI staining (*left* panel). The data are shown graphically with statistical analysis (*right* panel). The data are shown as mean ± SD (*n* = 3). *p <0.05, **p <0.01 compared with A3.01 cells. **b** Western blotting was used to determine the levels of expression of p53 and p-p53 using appropriate antibodies under same conditions

Anticancer drugs effectively promote cell apoptosis linked to genotoxic stress inducing p53 activation. Given this background, we sought to determine whether other types of anticancer drugs can selectively induce p53-mediated apoptosis of cells latently infected with HIV-1. Anticancer drugs including etoposide and doxorubicin, widely used in cancer therapy, were used to treat p53 competent ACH2 and NCHA2 cells as well as p53 null J1.1 cells. In an analysis of sub-G1 DNA content, the sub-G1 population of ACH2 and NCHA2 cells was markedly increased in comparison with uninfected A3.01 cells after etoposide or doxorubicin treatment similarly with 5-FU treatment. Unlike p53 competent cells, p53 null J1.1 cells showed slight resistance to apoptosis (Fig. 3a). The p53 phosphorylation levels were greatly increased in latently infected ACH2 and NCH2 cells in the presence of anticancer drugs in comparison with uninfected cells, whereas J1.1 cells did not show p53 expression after any drug treatment (Fig. 3b). These findings suggest that apoptosis of cells latently infected with HIV-1 might be induced by widely used anticancer drugs via p53 activation.

### p53 plays a pivotal role in the anticancer drug-induced increased apoptosis of cells latently infected with HIV-1

**Fig. 4:**
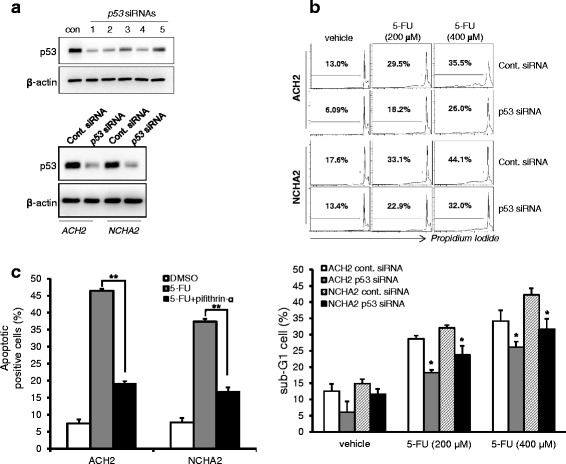
p53 knockdown diminished the death of cells latently infected with HIV-1. **a** Five p53 siRNAs were individually introduced into A3.01 cells using electroporation. After 48 h, Western blotting was used to determine the levels of expression of p53 (*upper* panel). Western blotting was used to determine the knockdown effect of p53 siRNA (No. 1) in latently infected cells (ACH2 and NCHA2) upon 5-FU treatment (*lower* panel). **b** The p53 in these cells was knocked down with p53 siRNA (No. 1) were treated with 5-FU (200–400 μM) for 36 h. The sub-G1 population was determined using flow cytometry. Total apoptotic cells are shown graphically with statistical analysis. The data are shown as mean ± SD (*n* = 3). *p <0.05 compared with cells electroporated with con. siRNA cells. **c** The cells were treated with 5-FU in the presence or absence of pifithrin-α for 48 h, and the apoptosis levels were then determined using flow cytometry. Apoptotic cells are shown graphically with statistical analysis. The data are shown as mean ± SD (*n* = 3). **p <0.01 compared with cells treated with the vehicle

To determine whether p53 plays a key role in the anticancer drug treatment-induced apoptosis of cells latently infected with HIV-1, siRNA against p53 was introduced into latently infected ACH2 and NCHA2 cells. The levels of expression of p53 were efficiently diminished in cells electroporated with No.1 p53 siRNA (Fig. 4a upper), and the p53 knockdown with the chosen siRNA was efficient in ACH2 and NCHA2 cells even upon 5-FU treatment (Fig. 4a lower). The knockdown of p53 by the siRNA significantly reduced the sub-G1 populations of latently infected ACH2 and NCHA2 cells in the presence of 5-FU (Fig. 4b), which may conceivably be the result of disruption of the p53 downstream pathway, triggering cell apoptosis. The role of p53 in the apoptosis of these cells upon 5-FU treatment was further evidenced with the p53 inhibitor. As shown in Fig. 4c, pifithrin-α treatment diminished cell apoptosis in both the ACH2 and NCHA2 cells upon 5-FU treatment. These data suggested that after anticancer drug treatment, the latently infected cells were sensitive to apoptosis via a p53 linked-pathway.

## Discussion

During the clinical course of HIV-1 infection, latently HIV-1 infected provirus is present in the long-lived CD4+ T memory cells that play a role as a steady source of infectious viral particles after interruption of treatment and as a major obstacle to HIV-1 eradication [4, 5]. Eliminating such HIV-1 reservoirs is a great challenge, because a selective marker by which to target the reservoir latently infected with HIV-1 has not yet been discovered.

HIV-1 infection can facilitate p53 expression and activation in T cells by integration-mediated host chromosomal abnormality and/or Vpr-induced DNA damaged-stress and Vpu-mediated stabilization of p53 [8, 10, 11, 23]. The induced p53 may play roles in the host restriction of HIV-1 replication and in the apoptosis of cells acutely infected with HIV-1. Anticancer drugs such as 5-FU, doxorubicin, and etoposide are well known to induce genotoxic stress linked to p53 activation followed by cell apoptosis. Although important roles for p53 in HIV-1 infection have been considered, utilization of anticancer drugs to activate p53 and selectively eliminate cells latently infected with HIV-1 has not been addressed.

In the present study, 5-FU induced the early and late apoptosis of latently infected ACH2 cells compared with latently infected J1.1 and uninfected A3.01 cells. Moreover, the levels of intracellular apoptotic markers such as cleaved caspase-3 and PARP were markedly increased in ACH2 cells compared with those in other cells (Fig. 1). The uninfected A3.01 cells that is p53 competent also showed significantly increased apoptosis upon 5-FU treatment compared with that of p53 defective J1.1 cells, while the levels were lower than those of the latently infected ACH2 cells derived from A3.01 cells. These data reveal that the differences in status of p53 among uninfected and latently infected cells (Fig. 2a) may lead to p53 dependent tendencies toward apoptosis upon genotoxic stress (Figs. 1, 2 and 3). Larrosa et al. showed that H9, J1.1, and U1 cells latently infected with HIV-1 were resistant to cytotoxic substances such as H_2_O_2_ and STP when compared with their uninfected parent cells [13]. However, their findings may do not support the resistance to apoptosis of latently infected cells in general, because the cells they used were all p53 null cell lines, which showed resistance to apoptosis upon substance-induced stress. Consistent with a previous report, our data also showed that p53 null J1.1 cells were more resistant to apoptosis upon genotoxic stress than uninfected A3.01 (Figs. 1 and 3) and Jurkat cells (data not shown). Together, these data indicate that latent HIV-1 infection may prevent apoptosis of the host cell to promote longer survival of the cells by an as yet unknown mechanism in the absence of p53, but HIV-1 infection may facilitate the apoptosis of cells susceptible to HIV infection that have competent p53. Although p53 is known to be a key factor in inducing apoptosis in anticancer drug treated cells, the p53 null J1.1 cells still showed small amounts of cell death. These data indicated that the cells were undergoing apoptosis by the p53-independent pathway such as p73 [24], Bid [25], MCL2 [26] and ATM/E2F1 [27] that were showed in its parent Jurkat cells upon the genotoxic stresses.

Western blot analysis of p53 target gene proteins showed that the levels of Bax, puma, and p21, which are the best-known p53 targets, were greatly increased in latently infected cells expressing p53 upon 5-FU treatment. As such, these gene products may contribute to the sensitivity of cells to apoptosis. Although the 5-FU treated A3.01 cells and the J1.1 cells expressed with ectopic p53 also showed increased levels of apoptosis compared with the untreated cells, the levels were much weaker than those of the p53 competent cells infected with HIV-1. The data indicated that the insufficient level of p53 activation and the alternative pathway of p53 could not greatly increase apoptosis, as shown in the ACH2 and NCHA2 cells. CD4+ T lymphocyte depletion linked to HIV-1 infection has been explained by numerous mechanisms including abortive infection-induced pyroptosis [28], DNA-dependent protein kinase [29], Fas-ligand [30], p53 activation [8] and viral proteins Nef and Vpr [31, 32]. These observations sufficiently explained the apoptosis as a result of acute HIV-1 infection. Genini et al. suggested that activation of p53 by acute HIV-1 infection may contribute to the depletion of HIV-1 infected T cells [8]. The present data also showed that latently infected cells, which are p53 competent, showed an increased level of phosphorylated p53 and its target genes in the absence of drugs, similarly to acute infection. However, the slight enhancement of p53 activation in the latently infected cells might not be sufficient to trigger apoptosis. Accordingly, genotoxic stress induced by anticancer drugs promoted the p53 activation synergistically leading to apoptosis of latently infected cells.

Numerous anticancer drugs have been developed and used for the cure of cancerous disease. Among them, 5-FU, an inhibitor of thymine synthetase, doxorubicin, an inhibitor of topoisomerase II, and etoposide, an inhibitor of topoisomerase I, are widely used in the treatment of cancers [33]. Although the drugs act on different enzymes linked to chromosomal DNA, these drugs commonly induce DNA stress followed by p53 activation-induced apoptosis. Based on the mechanisms of action of these drugs, 5-FU and doxorubicin/etoposide efficiently induced p53 activation in latently infected cells, followed by apoptosis, but not in p53 null J1.1 cells (Figs. 1 and 3). A new generation of anticancer drug, such as taxane, which inhibit the microtubule synthesis during mitosis unlinked to DNA-damaging stress, are used widely [34]. Therefore, p53-mediated apoptosis by the drug should determine whether the drug selectively induces the apoptosis of latently infected cells.

To determine the exact role of p53 in apoptosis of latently infected cells upon treatment with an anticancer drug, p53 siRNAs and a p53 inhibitor were employed. Both could reduce the apoptosis of the cells upon 5-FU treatment. The diminished apoptosis in the ACH2 and NCHA2 cells by p53 siRNA and the inhibitor indicated that p53 played an important role in the anticancer drug-induced apoptosis (Fig. 4). However, the siRNA and the inhibitor could not completely reduce the apoptosis. The results might have been caused by the p53-independent pathways and the incomplete efficacy of the siRNA and the inhibitor. A mechanism underlying viral reactivation in response to anticancer drugs has been proposed [14]. However, the data presented did not show distinctive apoptotic fates between cells because they focused on viral reactivation in the reservoir cells during anticancer drug-induced apoptosis without comparison to uninfected parent cells or assessing p53 status.

Although several methodologies for isolation of latently infected cells ex vivo have been developed, studying the functional role of those cells is so far difficult. There are lot of hurdles, such as the rarity of reservoirs (<1 per 10^6^ CD4+ T cells), the absence of suitable markers by which to isolate cells and T-cell activator treatments for surmounting the rarity, which may activate a variety of cellular signals [35]. Therefore, evaluation of p53-mediated apoptosis of latently infected cells in vivo and ex vivo remains for further study.

## Conclusions

We conclude that anticancer drugs may be useful agents for selectively purging HIV-1 reservoirs and might contribute to insights for future therapeutic strategies to promote complete viral eradication.
